# Effects of multimorbidity coexistence on the risk of mortality in the older adult population in China

**DOI:** 10.3389/fpubh.2023.1110876

**Published:** 2023-04-05

**Authors:** Zhili Su, Li Huang, Jinghui Zhu, Shichen Cui

**Affiliations:** School of Public Health and Management, Wenzhou Medical University, Wenzhou, China

**Keywords:** elderly, multimorbidity coexistence, mortality, risk, cohort study

## Abstract

**Background:**

Multimorbidity coexistence is a serious public health issue affecting a significant number of older adults worldwide. However, associations between multimorbidity and mortality are rarely studied in China. We assessed the effects of multimorbidity coexistence on mortality among a nationwide sample of older adults from China.

**Objective:**

We analyzed 10-year (2008–2018) longitudinal data of 12,337 individuals who took part in China, a nationwide survey of people aged 65 years and above. We used the Cox proportional hazard model to determine the effects of multimorbidity on the all-cause mortality risk. We also examined mortality risk between sex and age obtained through differential analysis.

**Results:**

At baseline, 30.2, 29.9, and 39.9% of participants had 0, 1, and 2 or more diseases, respectively. The cumulative follow-up of this study was 27,428 person-years (median follow-up = 2.7 years; range, 0.01–11.3 years), with 8297 deaths. The HRs (95% CIs) for all-cause mortality in participants with 1, and 2 or more conditions compared with those with none were 1.04 (0.98, 1.10) and 1.12 (1.06, 1.18), respectively. The heterogeneity analysis indicated that, the mortality risk for 80–94 years and 95–104 years group with multimorbidity coexistence is 1.12 (1.05–1.21) and 1.11 (1.01–1.23), respectively, but the mortality risk for 65–79 years group with multimorbidity coexistence was not statistically significant. The heterogeneity analysis indicated that, the mortality risk for men and women in older adults with multimorbidity coexistence is 1.15 (1.06, 1.25) and 1.08 (1.01, 1.17), respectively.

**Conclusion:**

Multimorbidity coexistence is associated with an increase in an increased risk of death in older individuals, with the effect being relatively significant in those aged 80–94 years.

## 1. Introduction

According to the report in 2009, there were 113.09 million people aged 65 and above in China, accounting for 8.5% of the total population. By 2020, there will be 190.64 million people aged 65 and over in China, accounting for 13.5% of the total population (1). It means that the aging society is growing rapidly. The phenomenon of aging has led to a significant increase in chronic diseases, which in turn has led to an increase in the incidence of multimorbidity. Multimorbidity coexistence is the presence of two or more adverse health conditions or diseases occurring together in an individual (2–4). Multimorbidity is now a major public health problem worldwide, and its coexistence leads to high mortality rates, reduced physical functioning, disability, and poor quality of life, adding to the heavy burden on healthcare systems (5–9). A study found that 75% of individuals aged 65–74 years had multimorbidity, with the proportion increasing to 80% in those aged 75 years and older (10). Although multimorbidity coexistence has been recognized, it has still not been adequately studied. Most previous studies were cross-sectional and showed a correlation between multimorbidity and functional status; however, most studies had relatively small samples (11).

Therefore, this study systematically explores the impact of multimorbidity on mortality risk in older people through a prospective cohort study, filling a data gap in this area of multimorbidity and providing a scientific basis for developing prevention and control strategies for healthy aging management.

## 2. Data and methods

### 2.1. Data and sampling

This study used the dataset from the 2008–2018 Chinese Longitudinal Healthy Longevity Survey (CLHLS).

The CLHLS is a longitudinal survey of a nationally representative sample of Chinese people aged 65 and older with accurate quality (12).

The survey covered 23 provinces in China and collected extensive data on a large population of the oldest old individuals aged 80–112 and comparatively younger elderly individuals aged 65–79 to serve the needs of scientific research on the elderly people.

**Figure 1:**
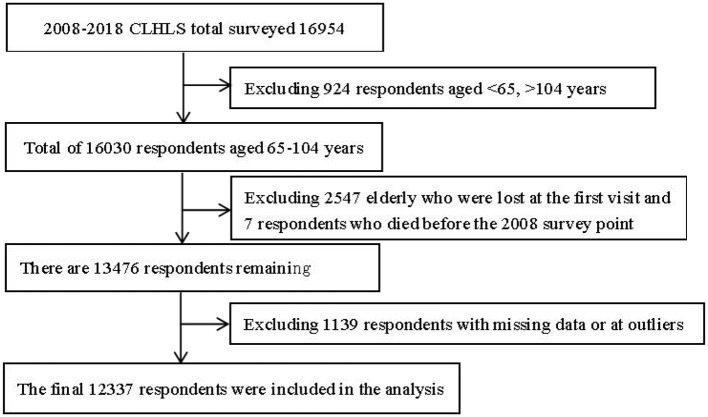
Flow chart for the inclusion of respondents.

The 2008 survey was used as the baseline survey for this study, which included four periods of longitudinal data from 2008, 2011, 2014, and 2018. Exclusion criteria were as follows: first, 924 respondents who were younger than 65 and older than 104 years old at the baseline survey (2008) were excluded (13). Second, 2,554 respondents who were lost at the first follow-up and died before the 2008 survey time point were excluded. Finally, 1,139 respondents with missing data or outliers were excluded. The final sample of this study comprised 12,337 respondents with valid information. The flow chart is shown in Figure 1.

### 2.2. Multimorbidity assessment

In this study, elderly people with two or more diseases were classified as multimorbidity coexistence according to the method commonly used in previous studies (2–4). The diseases in this study are based on the CLHLS 2008 disease survey, in which respondents answered 19 chronic diseases collected from self-reported: hypertension, diabetes, heart disease, stroke and cerebrovascular, gastrointestinal ulcers, Parkinson's disease, bedsores, arthritis, dementia, epilepsy, cholecystitis, dyslipidemia, chronic nephritis, hepatitis, asthma, tuberculosis, glaucoma, cataracts, and cancer. We categorized study participants according to multimorbidity number at baseline as having either no disease group, single disease group, or multimorbidity (two or more diseases) group.

### 2.3. Outcome assessment

The main outcome of this study was all-cause mortality. Mortality risk was assessed using survival status and survival time calculated from months lived from the baseline (2008) to 2018. Survival is from the start of the survey in 2008 until their death or the time of the last follow-up visit. Lost visits were those in which the person or the elderly person's family could not be contacted or dropped out. The main outcome was all-cause mortality occurring during the follow-up survey in 2008–2018, with followed up every 3 years.

### 2.4. Covariates

Although the focus of this article was on the effects of multimorbidity coexistence on mortality, it was important to control for other factors so that the results would be reliable. Demographic, lifestyle, and physiological health characteristics were obtained, including age, sex, urban/rural residence, mode of residence, years of education, financial status, marital status, smoking, drinking, physical exercise, the ability to daily living (ADL), cognitive health, sleep status, and body mass index.

Age was divided into three groups: 65–79, 80–90, and 95–104 years. Mode of residence was dichotomized as living with family and others. Marital status is “married living with a spouse,” while divorced, married not living with a spouse, widowed, and unmarried are classified as “other.” The economic situation according to the question “your life is compared locally, belong to” was divided into “well-off,” “average,” and “poor.” Current smoking, current alcohol drinking, and regular physical exercise were dichotomized as yes vs. no. Difficulty with ADLs is commonly used to gauge older people's daily performance in six basic activities: bathing, dressing, eating, using the toilet, free movement, and urine and defecation control. For ADL, a binary variable was constructed, with 1 representing having difficulty with any of the six ADLs and 0 representing having no difficulty with any of the six ADLs. Sleep duration was classified as <6 h, 6–10, and more than 10 h. BMI, defined as the weight (kg) in kilograms divided by the height (m) squared, were categorized as underweight (< 18.5), normal weight (18.5–23.9), and overweight or obese (≥24).

### 2.5. Statistical analysis

SPSS 27.0 software was used for data processing and statistical analysis in this study. Pearson's chi-square test was used for difference analysis between the groups. Kaplan–Meier survival curves demonstrate the risk of death for older people with different disease states, and log-rank tests are used to test for differences between groups. Cox proportional hazard regression was conducted longitudinally to investigate the relationship between multimorbidity and mortality risk. In addition, the relationship between multimorbidity and mortality was explored based on stratifying age and sex. The hazard ratios (HRs) for all-cause mortality with 95% confidence intervals were expressed for the results. All statistical analyses were performed using a two-sided test with a test statistic of *a* = 0.05.

## 3. Results

### 3.1. Descriptive statistics

This study comprised a total of 12,337 participants at baseline. At the time of the survey, the M (P25, P75) was 87 (77, 94) years; 55.3% were female subjects, 61.7% were uneducated, and 63.3% lived in rural areas. Of these, 3,732 (30.2%), 3,686 (29.9%), and 4,919 (39.9%) were categorized as having no, single disease, and multimorbidity (two or more diseases), respectively. More than half of the participants with death were 80–94 years and female, rural, uneducated, ADL normal, and had multimorbidity (two or more diseases). The baseline characteristics of the survey respondents are shown in Table 1. There were statistical differences (*p* < 0.05) in age, residence, education, marital status, smoking, drinking, physical exercise, BMI, sleep duration, financial status, and ADL when comparing the deceased and surviving groups.

**Table 1:** Comparison of baseline characteristics of study participants by follow-up outcome, 2008–2018 (%).

**Variables**	**Total (12,337)**	**Death (8297)**	**Survival (4040)**	**χ^2^**	***P*-value**
Age (years)				3121.3	<0.001
65–79	3,632 (29.4%)	1,160 (14.0%)	2,472 (61.2%)		
80–94	5,765 (46.7%)	4,429 (53.4%)	1,336 (33.1%)		
95–104	2,940 (23.8%)	2,708 (32.6%)	2,32 (5.7%)		
Gender				3.06	0.08
Male	5,520 (44.7%)	3,667 (44.2%)	1,853 (45.9%)		
Female	6,817 (55.3%)	4,630 (55.8%)	2,187 (54.1%)		
Residence				56.5	<0.001
Urban	4,523 (36.7%)	2,853 (34.4%)	1,670 (41.3%)		
Rural	7,814 (63.3%)	5,444 (65.6%)	2,370 (58.7%)		
Living style				0.1	0.932
Living with family	10,182 (82.5%)	6,846 (82.5%)	3,336 (82.6%) 704		
Other	2,155 (17.5%)	1,451 (17.5%)	(17.4%)		
years of education				393.5	<0.001
≥1	4,728 (38.3%)	2,677 (32.3%)	2,051 (50.8%)		
0	7,609 (61.7%)	5,620 (67.7%)	1,989 (49.2%)		
Marital status				1130.7	<0.001
living with spouse	4,094 (33.2%)	1,928 (23.2%)	2,166 (52.9%)		
Other	8,243 (66.8%)	6,369 (76.8%)	1,874 (46.4%)		
Smoking				30.5	<0.001
No	1,0021 (81.2%)	6,852 (82.6%)	3,169 (78.4%)		
Yes	2,316 (18.8%)	1,445 (17.4%)	871 (21.6%)		
Drinking				19.8	<0.001
No	10,068 (81.6%)	6,861 (82.7%)	3,207 (79.4%)		
Yes	2,269 (18.4%)	1,436 (17.3%)	833 (20.6%)		
Exercise				277.6	<0.001
No	8,778 (71.2%)	6,297 (75.9%)	2,481 (61.4%)		
Yes	3,559 (28.8%)	2,000 (24.1%)	1,559 (38.6%)		
BMI (kg/m^2^)				396.3	<0.001
18.5-23.9	6,354 (51.5%)	4,201 (50.6%)	2,153 (53.3%)		
< 18.5	3,984 (32.3%)	3,065 (36.9%)	919 (22.7%)		
≥24	1,999 (16.2%)	1,031 (12.4%)	968 (24.0%)		
Sleep status (hours)				211.3	<0.001
6-10	9,631 (78.1%)	6,273 (75.6%)	3,358 (83.1%)		
< 6	1,496 (12.1%)	985 (11.9%)	511 (12.6%)		
> 10	1,210 (9.8%)	1,039 (12.5%)	171 (4.2%)		
Economic status				22.1	<0.001
Wealthy	1,654 (13.4%)	1,072 (12.9%)	582 (14.4%)		
Average	8,455 (68.5%)	5,637 (67.9%)	2,818 (69.8%)		
Poor	2,228 (18.1%)	1,588 (19.1%)	640 (15.8%)		
ADL				300.5	<0.001
Normal	9,067 (73.5%)	5,699 (68.7%)	3,368 (83.4%)		
Disorders	3,270 (26.5%)	2,598 (31.3%)	672 (16.6%)		
Morbidity				123.5	<0.001
No disease (0)	3,732 (30.2%)	2,283 (27.5%)	1,449 (35.9%)		
Single disease (1)	3,686 (29.9%)	2,451 (29.5%)	1,235 (30.6%)		
Multimorbidity (≥2)	4,919 (39.9%)	3,563 (42.9%)	1,356 (33.6%)		

### 3.2. Mortality

During the 10-year follow-up, out of the 12,337 participants gathered at baseline, 8,297 (67.3%) were reported deceased. The study had a cumulative follow-up of 27,428 person-years (median follow-up of 2.7 years), a cumulative mortality rate of 67.3%, and a mortality density of 302.5 deaths per 1,000 person-years. As shown in Table 2.

**Table 2:** Deaths during the follow-up of survey respondents.

**Grouping**	**Total number of people/occurrences**	**The cumulative follow-up person years**	**Cumulative mortality rate (%)**	**Death density/1000 person-years**
Total	12,337/8297	2,7428	67.3	302.5
Gender				
Male	5,520/3667	12,531	66.4	292.6
Female	6,817/4,630	14,897	67.9	310.8
Age (years)				
65–79	3,632/1,160	5,478	31.9	211.8
80–94	5,765/4,429	15,579	76.8	284.3
95–104	2,940/2,708	6,371	92.1	425.1
Morbidity				
No disease	3,732/2,283	8,239	61.2	277.1
Single disease	3,686/2,451	8,356	66.5	293.3
Multimorbidity	4,919/3,563	1,0832	72.4	328.9

### 3.3. Analysis of cox regression models of multimorbidity coexistence and mortality risk

Table 3 shows the HR of all-cause mortality according to multimorbidity. Regarding multimorbidity, those with no disease served as the reference group. Increased risk of mortality was significantly associated with multimorbidity. In Model 3, the HR was 1.04 (95% CI, 0.98–1.10) for one chronic disease and 1.12 (95% CI, 1.06–1.18) for multimorbidity.

**Table 3:** Cox proportional risk regression model for factors influencing death in older people.

**Morbidity**	**Model 1**		**Model 2**		**Model 3**	
	**HR (95% CI)**	***P-*** **value**	**HR (95% CI)**	***P-*** **value**	**HR (95% CI)**	***P-*** **value**
No disease	1.00		1.00		1.00	
Single disease	1.07 (1.01–1.13)	0.02	1.03 (0.98–1.10)	0.21	1.04 (0.98–1.10)	0.19
Multimorbidity	1.19 (1.13–1.26)	<0.001	1.10 (1.05–1.17)	<0.001	1.12 (1.06–1.18)	<0.001

HR, hazard ratio; 95% CI, 95% confidence interval. Model 1: corrected for sex, age, place of residence, and mode of residence; Model 2: further adjusted for education, marriage, smoking, alcohol consumption, exercise, economics, and ADL; Model 3: further adjusted for sleep, and BMI.

**Figure 2:**
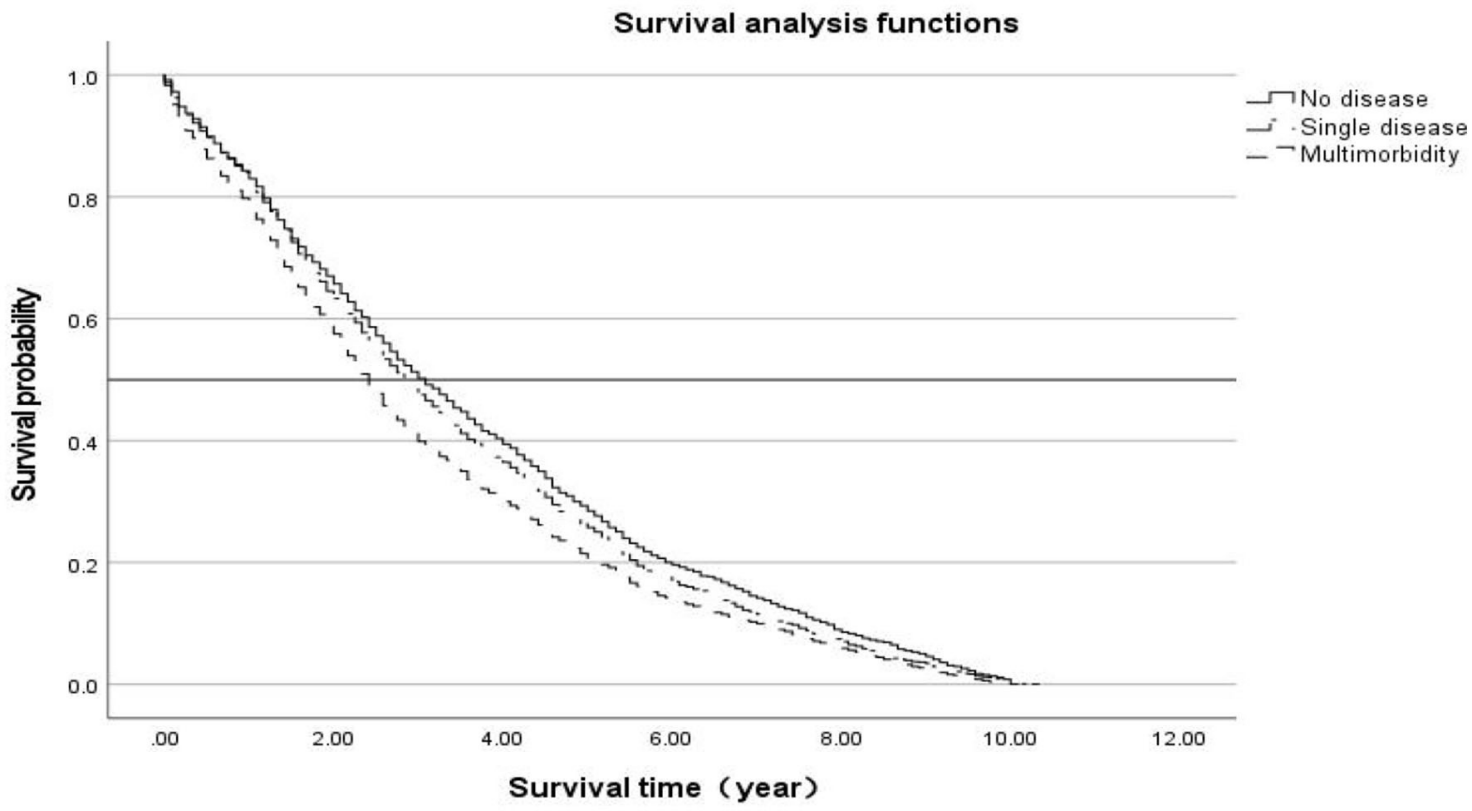
Kaplan–Meier survival curve.

The Kaplan–Meier survival curve (Figure 2) results showed that survival time M (P25, P75) was 2.7 (2.6, 2.7). Older people with multimorbidity had a higher mortality rate than those without disease, with a log-rank chi-square value of 69.4, *p* < 0.001, indicating a statistically significant difference.

### 3.4. Stratified analysis of the association between multimorbidity coexistence and mortality risk

Next, we examined the stratified analysis of age and gender on all-cause mortality. As shown in Table 4, concerning gender, we observed a positive association between gender and mortality in the fully adjusted model, with the HR for the risk of death from multimorbidity being 1.15 (95% CI, 1.06–1.25) for men and 1.08 (95% CI, 1.01–1.17) for women (*p* < 0.05) compared to the no disease group. For age, the HR for the risk of death due to multimorbidity was 1.12 (95% CI, 1.05–1.21) for 80–94 years old compared to the no disease group; the HR for the risk of death due to multimorbidity was 1.11 (95% CI, 1.01–1.23) for 95–104 years old (*p* < 0.05), but no differences in multimorbidity and mortality were observed by 65–79 years.

**Table 4:** Stratified analysis of multimorbidity coexistence and mortality risk in older people.

**Variables**	**HR**	**95% CI**	***P*-value**
Gender			
Male			
No disease	1.00		
Single disease	1.041	0.96–1.13	0.338
Multimorbidity	1.153	1.06–1.25	<0.001
Female			
No disease	1.00		
Single disease	1.028	0.95–1.11	0.504
Multimorbidity	1.084	1.01–1.17	< 0.032
Age (years)			
65–79			
No disease	1.00		
Single disease	1.115	0.96–1.29	0.14
Multimorbidity	1.083	0.94–1.25	0.28
80–94			
No disease	1.00		
Single disease	1.029	0.95~1.11	0.458
Multimorbidity	1.124	1.05~1.21	0.002
95–104			
No disease	1.00		
Single disease	1.021	0.92–1.14	0.71
Multimorbidity	1.108	1.01–1.23	< 0.045

Adjusted to Model 3.

## 4. Discussion

This study showed the findings of the survival analyses examining the effect of multimorbidity coexistence on all-cause mortality among older people of the CLHLS cohort over a 10-year follow-up period. Multimorbidity is defined by the presence of two or more long-term conditions, which are those that cannot currently be cured but can be controlled through medications or other treatments. Multimorbidity increases with age, and the majority of people with multimorbidity are older people (14). Multimorbidity coexistence is a relatively common phenomenon for older people. In this study, a total of 19 chronic diseases were investigated in the elderly people over 65 years of age, and the prevalence of multimorbidity was found to be 39.9%, which is higher than the national prevalence of 28.06% and is based on five common chronic diseases (hypertension, diabetes, chronic obstructive pulmonary disease, asthma, and tumors) (15).

This study found that individuals with multimorbidity are at an increased risk of all-cause mortality compared with those with no disease and a single disease. Multimorbidity is an independent factor that increases the risk of death, and it can also be a strong predictor of mortality. These results were significant even after having controlled for demographic characteristics, lifestyle, and physiological health, a finding that is consistent with other studies (16–18). The mortality risk increased for two or more chronic diseases at 1.12 compared to the no chronic disease groups. Basu's study showed that the higher the number of chronic diseases, the higher the degree of disability (19). Other studies have also shown that multimorbidity coexistence will lead to increased dementia, and frailty in the elderly people, affecting the overall physical function and quality of life, even produce significant financial burden on healthcare systems (20–22).

In addition, this study further explored the relationship between multimorbidity and mortality risk in older people by gender and age group. The results showed that the risk of death from multimorbidity coexistence was high in both male and female older adults and that this risk of death was more pronounced in male older adults. The discrepancy can be attributed to the high burden of unhealthy lifestyle factors in male subjects compared with female subjects, like most male subjects who smoke and drink alcohol, which in turn leads to a higher risk of death (23). This is consistent with the results of the analysis of chronic diseases based on Chinese death surveillance data from 2004 to 2018; the results show that at present and in future, the male age-standardized mortality rate is higher than that in female subjects (24). The age analysis found no differences in multimorbidity and mortality observed by 65–79 years old, compared to the no disease or single disease. The risk of death due to multimorbidity for 80–94 years old and 95–104 years old is statistically significant, with a significant risk of death among 80–94 years old compared to 95–104 years old. Similar to previous studies (25, 26), these findings showed that multimorbidity was more likely to occur among those of older age than those of younger age. This may be because the elderly people are gradually aging with the increase of age, with reduced immunity, and degenerative changes were observed in each system organ, resulting in the increased risk of suffering from multiple chronic diseases.

In summary, the accelerated effect of multimorbidity on elderly people's health, and the gender and age difference of the effect should cause great attention to all sectors of society. In the context of disease transition and aging society, multimorbidity coexistence has become a key factor hindering the improvement of the health level of elderly people in China, Older people “live long,” but it is necessary to make them “live well.” Currently, there is no effective treatment for multimorbidity and still needs frequent general practice consultations, complex and structured care, as well as coordination between health and social sectors (27). From the National Institute for Health and Care Excellence (NICE) record an approach to care that takes account of multimorbidity, involving a personalized assessment and the development of an individualized management plan (28). This study aimed to improve quality of life by reducing treatment burden, adverse events, and unplanned or uncoordinated care. Focusing on the quality of life and symptoms of patients with multimorbidity became the main target.

This study analyzed the effects of multimorbidity coexistence on the all-cause mortality risk. In addition, the relationship between multimorbidity and mortality was explored based on stratifying age and gender. Relevant conclusions can provide some reference for paying clinical attention to key risk groups and providing intervention measures in future. However, there are some shortcomings in this study. First, the study examines the number of diseases rather than the pattern of diseases, which needs to be supplemented by further research. Second, the data limitations of this study do not allow for a detailed examination of individual disease subsections. Finally, the data used for multimorbidity were self-reported by patients, which may underestimate the chronic disease status of the study population.

## 5. Conclusion

This study analyzed the effect of multimorbidity on all-cause mortality using data from a 10-year cohort of the CLHLS. This study showed that individuals with multimorbidity are at an increased risk of all-cause mortality compared with those diseases alone, especially in 80–94 years and male elderly adults. Therefore, patients with multimorbidity must be provided with integrated assessment and treatment.

## Data availability statement

Publicly available datasets were analyzed in this study. This data can be found at: Chinese Longitudinal Healthy Longevity Survey (CLHLS).

## Ethics statement

The studies involving human participants were reviewed and approved by the Biomedical Ethics Committee, Peking University (IRB00001052-13074). The patients/participants provided their written informed consent to participate in this study.

## Author contributions

ZS and LH designed the research and directed its implication. ZS prepared, analyzed the data, and drafted the manuscript. JZ and SC revised the manuscript together. All authors have read and approved the manuscript.
